# Early intervention of multiple home visits to prevent childhood obesity in a disadvantaged population: a home-based randomised controlled trial (Healthy Beginnings Trial)

**DOI:** 10.1186/1471-2458-7-76

**Published:** 2007-05-10

**Authors:** Li Ming Wen, Louise A Baur, Chris Rissel, Karen Wardle, Garth Alperstein, Judy M Simpson

**Affiliations:** 1 Health Promotion Service, Sydney South West Area Health Service, New South Wales, Australia; 2 Discipline of Paediatrics & Child Health, University of Sydney, Australia; 3 Community Paediatrics, Sydney South West Area Health Service, New South Wales, Australia; 4 School of Public Health, Faculty of Medicine, University of Sydney, Australia

## Abstract

**Background:**

Studies have shown that a proportion of children as young as two years are already overweight. This indicates that obesity prevention programs that commence as early as possible and are family-focused are needed. This Healthy Beginnings Trial aims to determine the efficacy of a community-based randomized controlled trial (RCT) of a home visiting intervention in preventing the early onset of childhood overweight and obesity. The intervention will be conducted over the first two years of life to increase healthy feeding behaviours and physical activity, decrease physical inactivity, enhance parent-child interaction, and hence reduce overweight and obesity among children at 2 and 5 years of age in the most socially and economically disadvantaged areas of Sydney, Australia.

**Methods/design:**

This RCT will be conducted with a consecutive sample of 782 first time mothers with their newborn children. Pregnant women who are expecting their first child, and who are between weeks 24 and 34 of their pregnancy, will be invited to participate in the trial at the antenatal clinic. Informed consent will be obtained and participants will then be randomly allocated to the intervention or the control group. The allocation will be concealed by sequentially numbered, sealed opaque envelopes containing a computer generated random number. The intervention comprises eight home visits from a specially trained community  nurse over two years and pro-active telephone support between the visits. Main outcomes include a) duration of breastfeeding measured at 6 and 12 months, b) introduction of solids measured at 4 and 6 months, c) nutrition, physical activity and television viewing measured at 24 months, and d) overweight/obesity status at age 2 and 5 years.

**Discussion:**

The results of this trial will ascertain whether the home based early intervention is effective in preventing the early onset of childhood overweight and obesity. If proved to be effective, it will result in a series of recommendations for policy and practical methods for promoting healthy feeding and physical activity of children in the first two years of life with particular application to families who are socially and economically disadvantaged.

## Background

### The issue

Overweight and obesity are responsible for a large proportion of morbidity and mortality among the adult population [1-3]. While overweight and obesity typically become more common and severe, and have their greatest impact, later in life, longitudinal studies have shown that they have even more significant adverse effects when acquired early in life [4,5]. The age of onset and severity of overweight and obesity in childhood is related to its persistence into adulthood, with obese children having at least a 25–50% risk of progressing to obesity in adulthood [6].

There is overwhelming evidence of increasing high prevalence of childhood overweight and obesity over the past two or three decades, in particular, among children aged 2 to 5 years internationally [7-9]. In Australia, a recently published study, from Wave 1 of the Longitudinal Study of Australian Children [10], involving 4983 children aged 4–5 years, showed that 15.2% were overweight and an additional 5.5% were obese, with children from lower socioeconomic backgrounds having higher prevalence rates. In addition, a study of young children aged 2–4 years in Melbourne and Sydney found that about one in six were overweight or obese [11].

The rising prevalence of childhood obesity, its appearance in early life and the modest impact of interventions aimed at children with existing overweight [12,13], underline the imperative for an effective, home-based approach to prevent overweight and obesity in early childhood. However, few systemic interventions aimed at preventing early childhood overweight and obesity have been implemented and evaluated [12,14]. Although existing infant nutrition programs address overweight and obesity to some extent [15,16], this has not been the main focus of such interventions to date and nor has this particular aspect been evaluated. In addition, there has been little research or practice in the area of physical activity promotion in infancy and early childhood [17]. As with adults, an explicit aim of increasing physical activity and decreasing sedentary behaviour is likely to be required if obesity prevention is to be addressed.

### The contributing factors

While underlying genetic factors may predispose to overweight and obesity, the overarching cause is chronic energy imbalance – excessive energy intake and inadequate energy expenditure [18]. Behavioural factors such as artificial feeding (bottle feeding), dietary fat intake, sweetened drink intake, family eating patterns, levels of physical inactivity and television viewing have been identified as major contributing factors for childhood obesity [12]. There is also some evidence that overweight and obesity are more prevalent in lower socio-economic groups [10,19]. This may be due to poorer dietary and physical activity behaviours that are associated with social disadvantage, or it may be associated with some other aspect of social disadvantage that is less well understood.

The evidence also suggests that sustained breastfeeding, the appropriate introduction of solids, the avoidance of the use of less healthy foods and sweetened drinks as a reward, a comfort or a "pacifier", a reduction in sedentary behaviour and the encouragement of physical activity can contribute to the prevention of overweight in early childhood [20,21].

However, focusing on diet and physical activity alone may not be sufficient to explain the development of overweight in early childhood. The context of the parent-child feeding interaction and the environmental context for energy expenditure need to be considered for obesity prevention to be maximally effective. Energy imbalance results from complex interactions and in children the role of parents is, of course, paramount.

### Infant feeding and awareness of childhood obesity

To deepen our understanding of mothers' attitudes, beliefs and experiences about the issues related to infant feeding, we conducted a study as part of the needs assessment for developing this program [22]. The study showed that while there was good overall awareness of the recommendations about the duration of breastfeeding and the introduction of solids, many mothers were not acting on these because of various misconceptions about milk supply, the effect of solids on sleep, interpretation of the baby's behaviour as an indicator of his or her readiness for solids, and a general lack of understanding of the possible consequences of not following infant feeding guidelines. The overwhelming amount of confusing and often conflicting information from a variety of professional and social sources led women to the conclusion to "do what works for us". Using food for rewards was a common experience for most mothers, and although there was agreement about the potential dangers and inappropriateness of this practice, this was outweighed by the convenience and practicality. Finally, the study concluded that most mothers did not even consider that the early onset of childhood obesity was an issue [22]. Other studies have shown that even parents of overweight children fail to recognize excess weight as a health issue for their children [23].

### Home visiting programs

Home visiting programs, which provide social support to first time mothers, have been established as effective interventions for improving the health and wellbeing of parents and children [24-27]. These programs have been widely promoted as a means of preventing a range of health and developmental problems in children from vulnerable and disadvantaged families. Research evaluating early intervention strategies highlights the need for programs such as home visiting to be a part of broader strategies for promoting healthy family functioning and social support [28]. Family functioning has been found to be determined by a range of factors including the quality of relationships, the health of family members and the presence of life stressors. Social support is determined by the availability of others to share problems and feeling close. Home visits provide the opportunity for parents to address family functioning problems with others as well as providing social support. To have lasting beneficial effects, Ramey and Campbell suggested that the intervention needs to start early, preferably before the child is born, and to continue beyond the first two years of the child's life, providing continuing support until the child starts school [28].

Home visiting has been adopted as one of the strategies in the NSW Families First initiative. Families First is the NSW Government's prevention and early intervention program to help parents give their children "a good start in life" [27]. Its main concerns are to improve the general health of mothers and babies and to build communities that work to support families. In NSW all new mothers are offered one home visit from Early Childhood Services within a month of the birth of a child. It is intended for vulnerable families to receive multiple home visits for at least two years.

### The home-based early intervention to prevent childhood obesity

In this proposed study we will implement and evaluate a structured intervention specifically addressing the issues of childhood obesity to be delivered by an early childhood health nurse during the first two years of life. The intervention has been developed and refined through a series of parallel processes. First, a pilot program has been completed with some promising results [29]. The program was very well received by the participating mothers, with a retention rate of 95% over a year. Compared to the state population average, the rate of breastfeeding at 12 months was significantly higher (36% vs 18%, χ^2^_1 _= 8.21, P < 0.01), there was a lower rate of the introduction of solids before 4 months (2% vs 13%, χ^2^_1 _= 8.73, P < 0.01), and there was a high rate of cup feeding at 12 months (98%).

Second, existing evidence of the effectiveness of interventions has also informed the development of this program, such as the lessons learned by various studies in working with families of overweight children, and the dynamics of child feeding [12,13,15,16]. In addition, health promotion theories, such as the Health Belief Model, have been applied in informing the development of the intervention to increase perceptions of susceptibility of children being overweight and social learning models including attention to social support and relapse prevention. The series of topic specific interventions has been planned using an eclectic theoretical approach based on all that is currently known about the dynamics of child feeding, other behaviours and their effect on weight.

## Aims and hypotheses

The **aim of the study **is to compare the effect of an intensive, home-based early intervention for first-time mothers with newborn babies, versus usual care, on child and family eating patterns, television viewing, opportunities for physical activity and anthropometric measures.

First-time mothers with a newborn will receive one of the two following interventions:

• Intensive home-based early intervention delivered by a specially trained nurse over the first two years of life, promoting healthy feeding and physical activity and enhanced parent-child interaction;

• Usual care (a current standard practice, one home visit by a community nurse within a month of birth plus possible baby clinic visits) plus home safety promotion materials.

We **hypothesise **that the home-based early Intervention, relative to usual care, will:

• lead to a significantly increased duration of breastfeeding and a later introduction of solids;

• lead to a significantly decreased intake of sweetened drinks and an increased intake of fruit and vegetables at age 2 years;

• lead to a decreased amount of television viewing at age 2 years;

• lead to an increased range of opportunities for active play at age 2 years;

• lead to improved nutrition and physical activity levels in mothers and families

• be acceptable to mothers and families;

• be feasible in an Australian community health setting;

• lead to a significantly reduced level of overweight and obesity in children at age 2, and 5 years (to be assessed in a later follow-up study).

## Design and methods

### Overall study design

**Figure 1:**
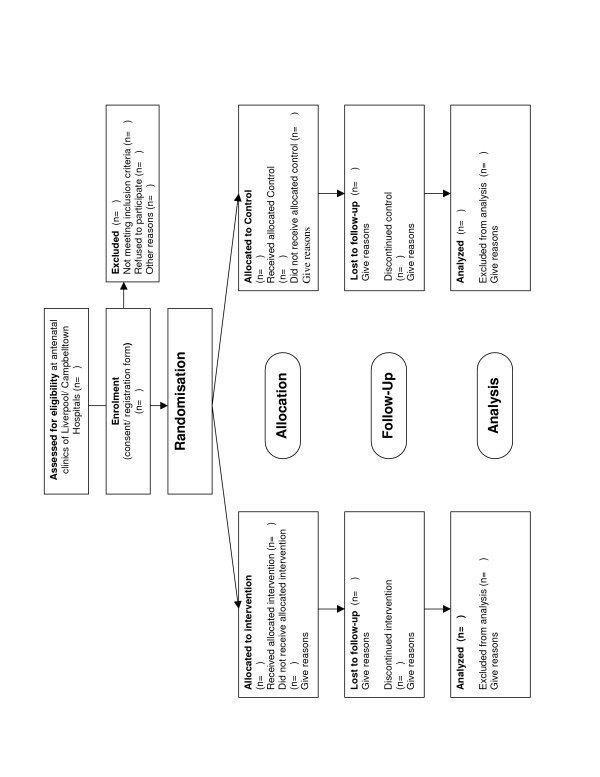
Study design.

The design of this proposed study is a randomised controlled trial (RCT) (see Figure 1). Two phases of the study are planned: an intervention phase for the first two years and a follow-up phase for the following three years. The current funding we have received covers the first two years of the study. Additional funding will be sought for further follow-up of participants, taking the total study period to 5 years. Ethical approval to conduct this trial has been granted by the Research Ethics Review Committee of the Sydney South West Area Health Service – Eastern Zone (ID number X04-0189).

### Participants and recruitment

All pregnant women who attend antenatal clinics of Liverpool and Campbelltown Hospitals will be approached by research nurses with a letter of invitation and information about the study. These two hospitals have been chosen since they attract a large number of patients from the areas of South West Sydney with the relatively high levels of disadvantage based on Social Economic Indexes For Areas (SEIFA) rankings [30] and a broad ethnic mix. Once eligibility is established and consent obtained, women will be asked to fill in a registration form with their contact information to allow the nurses to make further arrangement for the baseline data collection and random allocation to study condition. Mothers will only be entered into the study after written consent is obtained and eligibility criteria are met.

### Inclusion criteria

Women will be eligible to participate if they are aged 16 years and over, are expecting their first child, are between weeks 24 and 34 of pregnancy, are able to communicate in English and live in the local areas.

### Exclusion criteria

At the initial stage, women will be excluded from the study if they have a severe medical condition based on advice given by their doctors. Women who cannot give informed consent and those with known major fetal anomalies will also be excluded.

The further exclusion criteria after giving birth extend to:

• presence of a physical or intellectual disability in a child which is likely to influence physical activity or food habits;

• presence of a chronic health problem in a child likely to influence weight or height;

• inability of parents or carers to communicate in spoken English.

### Sample size

A consecutive sample of 782 first time mothers (391 in each arm of the study) will be required for the study. The sample size calculation is based on detecting changes in the main outcomes: weight status and key behaviour factors. We selected the larger result from the following two calculations:

1. To detect a difference in mean body mass index (BMI) z-score of 0.25 units between the groups at age 3 years a sample size of 252 for per group is needed. This was based on an estimated mean body mass index (BMI) of 16.8 and SD of 1.5 from an earlier pilot study of 174 children aged 3 years in Central Sydney [11].

2. One of the main predictors for childhood obesity is television-viewing time [31-34]. From previous studies we conservatively predict a 10% reduction in TV viewing after our intervention. A total sample size of 626 families (313 per arm) is required to have 80% power to detect a 10% difference between groups in the proportion of children watching more than 2 hours of TV/video per day at 2 years of age, at the two-sided 5% significance level. To allow for an estimated 20% drop out, we will recruit a total of 782 first-time mothers.

### Registration and randomisation

Information collected from the registration form will be entered into a password-protected Microsoft Access database. A letter will be sent by the research nurse to inform the hospital obstetrician that the mother is enrolled in this research trial. An appointment for the first home visit will be made with all participating mothers by a letter, followed by a telephone call. The first home visit will take place between weeks 30 to 36 of pregnancy.

Random allocation to either the intervention or control group is concealed by sequentially numbered, sealed opaque envelopes containing the group allocation, which will be determined by a computer generated random number. Randomisation will be stratified by hospital, with a block size of 50. A research assistant who has no direct contact with participating mothers will be responsible for generating the random numbers and preparing the envelopes.

During the first home visit, immediately after the baseline data collection, the nurse will open the sealed envelope and inform the mother of the outcome of randomisation.

### Intervention group

The intervention comprises eight home visits from a specially trained community nurse delivering the staged intervention, which includes one home visit at the gestation age of 30–36 weeks and seven visits at 1, 3, 5, 9, 12, 15 and 24 months after birth, together with pro-active telephone support. The timing of the visits corresponds to milestones in early childhood development, in particular with regard to healthy feeding practice, nutrition and physical activity as well as parent-child interactions (see Table 1).

**Table 1:** Stage and focus of the intervention

	Time of visit							
	
Focus of each visit	Before child's birth	At 1 month	At 3 months	At 5 months	At 9 months	At 12 months	At 15 months	At 24 months
Breastfeeding	+++	+++	+++	+	+	+/-		
Introduction of solids		++	+++	++	++	++		
Food texture and serving size				+	++	++	+++	+
Bottle vs cup feeding				+	++	+++	+	+++
Introduction of cow's milk		+	+	+	++	++	++	+++
Consumption of 'extra' foods		+	+	+	+	++	++	++
Healthy food choices (no food rewards)				+	+	++	++	+++
Parent-child interaction	+	+	+	+	+	+	+	+
Family and social support	+	+	+	+	+	+	+	+
Physical activity/Active Play		+/-	+	+	+	++	+++	+++
Sedentary behaviours eg TV viewing		+/-	+	+	+	++	++	+++

At each visit, the nurse will spend approximately one hour with the mother and infant, monitoring the parent-child feeding interaction and practice, and behaviours promoting physical activity/inactivity in the child. The nurse will identify the parental needs in relation to nutrition, feeding and age-appropriate physical activity/inactivity using a checklist. All information and recommendations provided to parents will be in keeping with National Health and Medical Research Council Dietary Guidelines [35], the Australian Guide to Healthy Eating [36] and the National Physical Activity Guidelines [37]. One-to-one consultation focusing on feeding behaviour and recommended problem-solving activities will be conducted. Pro-active telephone support will be provided between home visits to support behaviour maintenance and change. An individualised information kit will be provided to the parent.

### Control group

The control group families will receive the usual childhood nursing service from the Area Health Service, comprising one home visit by a community nurse within a month of birth plus possible baby clinic visits. However, for study participants in the control group there will be additional visits at baseline, 12 and 24 months, for the purpose of data collection only. To maximise the retention rate, we will also offer home safety promotion materials to the control group.

### Outcome measures

The proposed main outcome measures are set out in Table 2. Short questions about selected breastfeeding practices, food habits and physical activity levels will be used, which have been widely employed in population health surveys including the NSW Child Health Survey 2001 [38] and the Childhood asthma Prevention Study [39], and are supported by validity testing [40-42].

**Table 2:** Outcome measures for the study

**Phase One: intervention phase (0–2 years)**	**Phase Two: follow up phase (3–5 years)**
***Primary Measures***	
Duration of breastfeeding/timing of introduction of solids	
Intake of 'extra'^1 ^foods at age 2 years	Intake of 'extra' foods at age 3,4 & 5 years
Intake of fruit and vegetables at age 2 years	Intake of fruit and vegetables at age 3,4 & 5 years
Serving size at age 2 years	Serving size at age 3,4 & 5 years
Amount of television viewing at age 2 years	Amount of television viewing at age 3,4 & 5 years
Family nutrition and physical activity	Family nutrition and physical activity
Time spent undertaking active play at age 2 years	Time spent undertaking active play at age 3,4 & 5 years
Anthropometric measures of the child at age 2 years	Anthropometric measures of the child at age 3,4 & 5 years
***Secondary Measures***	
Parent-child interaction	
Parental knowledge/attitudes about childhood obesity	
Acceptability of the intervention to mothers and families	
Partner and family support	

^1 ^'Extra' foods include chips, wedges & crisps; takeaway burgers, pizza & chicken; sweetened drinks

In addition, two validation studies of measures of physical activity and nutrition for children aged 2–4 years are currently underway. These studies are being led by the NSW Centre for Public Health Nutrition, and the Discipline of Paediatrics & Child Health at the University of Sydney. The measurement tools will allow us to make detailed assessments of dietary intake and levels of physical activity and sedentary behaviour in order to detect the impact of the intervention.

Standard demographic and socio-economic information, and secondary measures will also be collected using questions from the NSW Child Health Survey 2001 [38].

Measurement points for both control and intervention groups for Phase One of the study will occur in participants' homes at baseline, 12 and 24 months. Phase Two measurements will take place at 3, 4 and 5 years.

Nurses trained in anthropometric techniques will collect measurements of height, weight and waist size in children at 2, 3, 4 and 5 years using stadiometers, scales and tape measures.

### Phase two study

Additional funding will be sought for a further follow-up of the cohort to assess the outcomes indicated above at 3, 4 and 5 years of age. This is an important dimension of the project, which will strengthen the overall study in the following ways. First, longer-term follow up will allow an assessment of whether any behavioural changes resulting from the intervention are sustained over time. Second, nutritional and physical activity behaviours in the first two years of life may not have a significant bearing on weight status during this immediate period. Finally, longer-term follow-up of the control group will permit prospective, observational analysis of the prevalence and determinants of overweight and obesity within a large, population-based sample during the early years.

### Data analysis

BMI will be calculated as weight (kg)/height (metres) squared and children at two to five years of age will be classified as not overweight or obese, overweight or obese, based on the International Obesity Taskforce recommended age-standardised BMI cut points [43].

The outcomes will be compared between the intervention and control groups. For continuous variables, such as knowledge and attitudes scales and BMI, means will be compared using t-tests, or non-parametric equivalents for non-normally distributed variables. For categorical variables, chi-squared tests will be used. All analyses will be by intention to treat.

## Discussion

Efforts to tackle childhood obesity must begin in early life. The epidemic is evident in children as young as two years of age. Attempts to treat existing obesity have had limited success. This may be due to the fact that established patterns of eating and physical activity behaviour are difficult to modify. Early life nutrition and physical activity levels are positively associated with obesity in childhood and later life. Moreover, parents with new-born children are very receptive to advice and support about lifestyle changes for the whole family from an early childhood health professional.

Few systemic interventions aimed at preventing early childhood overweight and obesity have been implemented and evaluated. This study will generate new evidence in the important area of childhood overweight and obesity prevention through a family based early intervention program. If successful, this study will also result in the development of a model of "best practice" for promoting healthy feeding and physical activity in infants under two years of age, which could be used nationally and internationally.

## Competing interests

The author(s) declare that they have no competing interests.

## Authors' contributions

LMW, LB, CR and GA conceived the project, and contributed to the development of the trial and the procurement of the funding. LMW drafted the manuscript. All authors commented on drafts and have approved final text.

## Pre-publication history

The pre-publication history for this paper can be accessed here:


